# Matteucci on Living Beings

**Published:** 1848-05

**Authors:** 


### MATTEUCCI ON LIVING BEINGS.

This is a highly attractive volume, the reprint of which we owe to the same house. It comprises a series of lectures on the *Physical Phenomena of Living Beings*, among which are many of the most interesting known to philosophy. We regret that we cannot find room in this number for a full notice of the work, but we hope to be able to do so at an early period.

